# Symptomatic renal papillary varicosities and medullary nephrocalcinosis

**DOI:** 10.1186/s12894-021-00931-3

**Published:** 2021-11-29

**Authors:** Brent Cleveland, Michael Borofsky

**Affiliations:** University of Minnesota, 420 Delaware St. S. E., MMC 394, Minneapolis, MN 55455 USA

**Keywords:** Renal papillary varicosity, Renal vascular malformation, Nephrocalcinosis, Essential hematuria, Laser fulguration, Case report

## Abstract

**Background:**

Nephrocalcinosis is often asymptomatic but can manifest with renal colic or hematuria. There is no reported association between nephrocalcinosis and renal vascular malformations, which may also be a source of hematuria. We herein present a case of a patient with hematuria related to nephrocalcinosis and renal papillary varicosities. These varicosities were diagnosed and successfully treated with flexible ureteroscopy and laser fulguration.

**Case presentation:**

A 24-year-old female with a history of epilepsy (on zonisamide), recent uncomplicated pregnancy, and new diagnosis of nephrocalcinosis presented with right flank pain and intermittent gross hematuria. Imaging revealed intermittent right sided hydronephrosis. A cystoscopy identified hematuria from the right ureteral orifice. Diagnostic flexible ureteroscopy revealed numerous intrapapillary renal stones and varicose veins of several renal papillae. A 200 μm holmium laser fiber was used to unroof these stones and fulgurate the varicosities with resolution of her symptoms for several months. She later presented with left-sided symptoms and underwent left ureteroscopy with similar findings and identical successful treatment.

**Conclusion:**

Unilateral hematuria from discrete vascular lesions of the renal collecting system may be obscured by other benign co-existing conditions, such as nephrocalcinosis and nephrolithiasis. Although a simultaneous presentation is rare, flexible ureteroscopy with laser fulguration offers an ideal diagnostic and therapeutic modality for these concurrent conditions if symptoms arise.

## Background

Nephrocalcinosis describes a condition in which calcium oxalate or calcium phosphate deposition occurs within the renal parenchyma. It is often asymptomatic and discovered incidentally on radiographic imaging. When symptoms do occur, they often include renal colic from associated nephrolithiasis or extrusion of calcium deposits into the collecting system, both of which can result in hematuria [1].

There is no reported association between nephrocalcinosis and renal vascular malformations. Although rare bilateral cases have been reported, generally these malformations are unilateral sources of hematuria. When diagnosed endoscopically, these vascular abnormalities have been classified as either discrete or diffuse lesions. Benign discrete lesions include renal hemangioma, minute venous rupture, or previously unidentified renal calculi. Although varices of the renal papillae have been categorized with MVR, there is a paucity of literature describing this unique clinical entity and its management. After obtaining informed voluntary consent from the patient, herein we describe a case of the incidental diagnosis and successful treatment of symptomatic varicosities of renal papillae in the setting of medullary nephrocalcinosis and recent pregnancy.

## Case presentation

A 24-year-old female with a history of epilepsy (on zonisamide) and an uncomplicated pregnancy with spontaneous vaginal delivery 1 week prior presented with right flank pain and intermittent gross hematuria. Her work-up was concerning for pyelonephritis, and CT imaging revealed right hydronephrosis as well as findings consistent with medullary nephrocalcinosis. The hydronephrosis was suspected to be secondary to her recent pregnancy; however, due to concern for possible urosepsis she underwent multiple attempts at renal decompression, including placement of 2 ureteral stents and a percutaneous nephrostomy tube, all which were dislodged. Of note, during the second ureteral stent placement, right distal ureteroscopy was performed and identified blood clot within the right ureter. She was treated with culture directed antibiotics but re-presented to a tertiary center with recurrent fevers. Repeat CT imaging demonstrated persistent right hydronephrosis. Ultimately, she declined another surgical intervention and elected for conservative management with additional antibiotics.

**Fig. 1:**
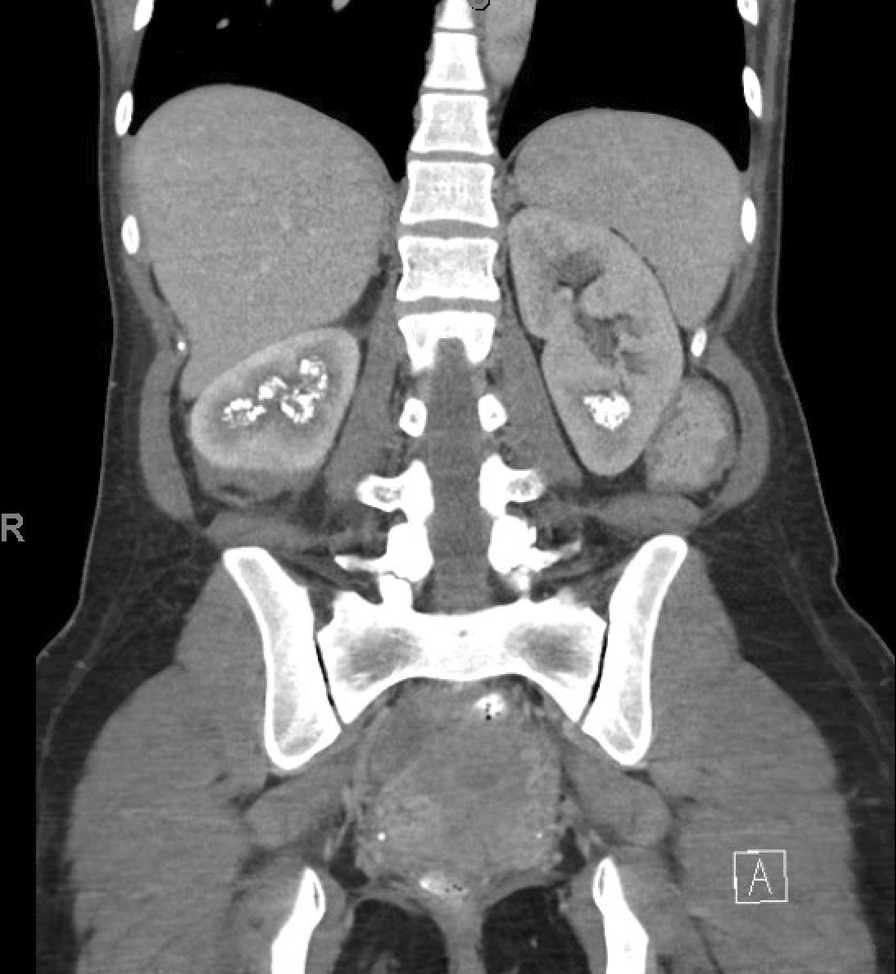
Coronal CT image demonstrating patient’s focal nephrocalcinosis prior to treatment

She was later seen on an outpatient basis at which time she reported intermittent right flank pain and gross hematuria, including string-like clots suspicious for upper tract bleeding. Physical exam revealed inconsistent right CVA tenderness. Initially, a renal ultrasound demonstrated resolution of her right hydronephrosis. Over the course of several months she visited the hospital several times for these symptoms. During one such visit, repeat CT imaging showed recurrent right hydronephrosis and proximal hydroureter suggesting new obstruction. It also demonstrated stable bilateral focal nephrocalcinosis in the right upper and mid poles as well as the left lower pole (Fig. 1). Laboratory work-up included normal serum creatinine (1.02 mg/dL), bicarbonate (25 mmol/L), calcium (8.7 mmol/L), and intact parathyroid hormone (22 pg/mL). Urinalysis confirmed gross hematuria with > 182 RBC per high power field. A urine culture was negative for significant pathogenic bacterial growth. A 24-h urine collection revealed borderline low urine volume (1.89 L), normocalciuria (154 mg/day), hypocitraturia (103 mg/day), hyperoxaluria (48 mg/day), hypernatriuria (253 mmol/day), and elevated urine pH (6.9). Her zonisamide was identified as a potential contributing factor to her nephrocalcinosis, as it has been known to be associated with pharmacologically induced renal tubular acidosis, a pathophysiology capable of causing nephrocalcinosis and stone formation. The patient’s Neurologist therefore transitioned her to lamotrigine for her history of epilepsy.

**Fig. 2:**
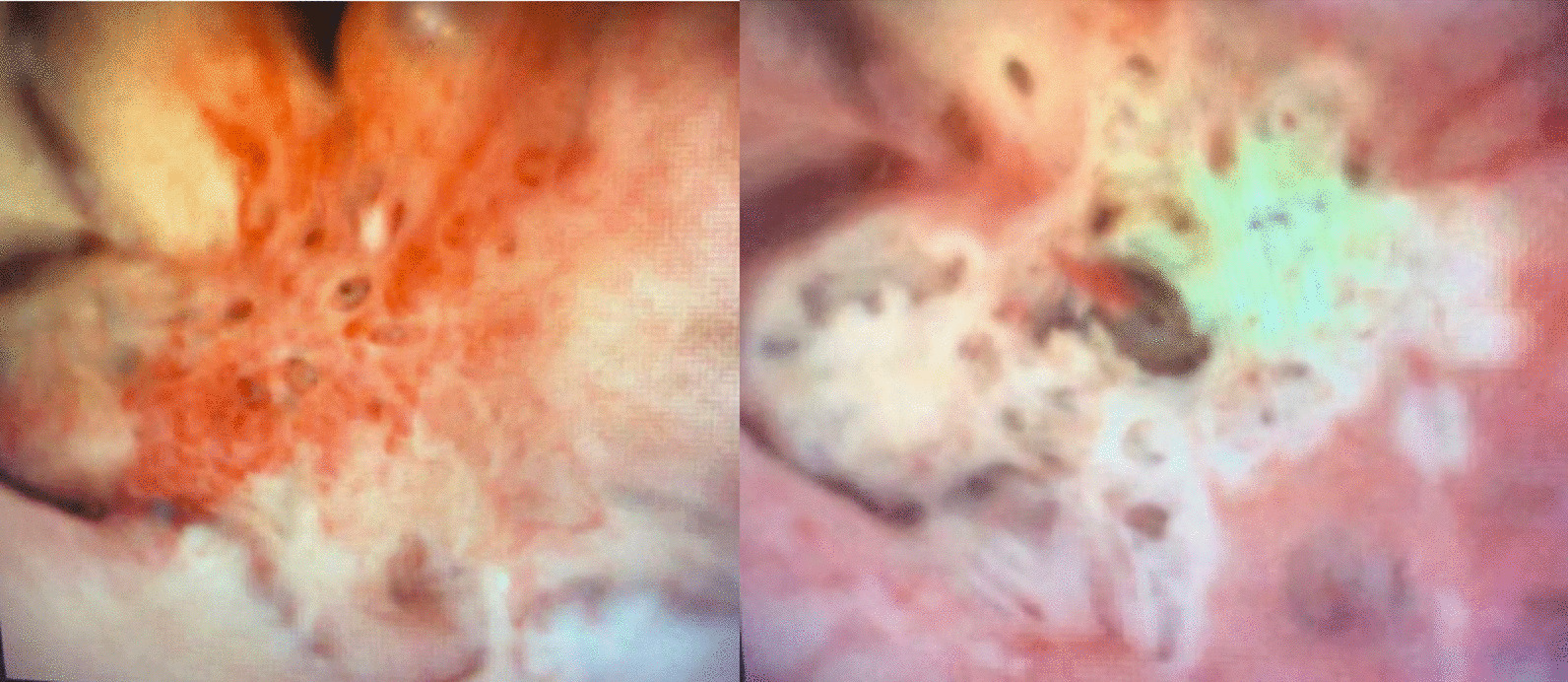
Intra-operative photo renal papillary varices before (left) and after (right) laser fulguration

After discussion of further diagnostic and management options, the patient elected for diagnostic right ureteroscopy. Her cystoscopy was unremarkable. Right retrograde pyelogram confirmed mild right hydronephrosis but showed no filling defects. Right ureteroscopy identified no ureteral lesions but did reveal numerous intrapapillary renal stones in the upper and mid poles of the right kidney with associated varicose veins of the renal papillae (Fig. 2). A 11/13 Fr 36 cm ureteral access sheath was placed under fluoroscopic guidance. A 200 μm holmium laser fiber was introduced and on the settings of 0.8 J and 8 Hz several embedded stones estimated to be < 2 mm in size were unroofed from the papillae. Next, using the laser settings of 0.4 J and 40 Hz on a long pulse mode the varicose veins were laser fulgurated (Fig. 2). Finally, a 6 Fr × 26 cm double J ureteral stent was placed on the right. She was observed overnight to monitor for hematuria. Her ureteral stent was dislodged overnight. She experienced no clinically significant bleeding. Her pre-operative and post-operative hemoglobin values were 12.7 and 12.2, respectively. She was discharged home the day after her surgery.

The patient was seen virtually for follow up 6 weeks later at which point she reported intermittent passage of tiny stones and complete resolution of her hematuria. A repeat renal ultrasound 9 weeks post-operatively demonstrated no hydronephrosis. A stone composition analysis revealed her renal stones were 10% calcium oxalate and 90% calcium phosphate.

She returned with left flank pain and gross hematuria about 10 weeks after her right sided treatment. CT imaging revealed a left ureteral stone and nonobstructing left renal calculi. Cystoscopy revealed isolated hematuria from the left ureteral orifice and a left sided ureteral stent was placed. She was transferred to a tertiary center where she underwent left diagnostic ureteroscopy. Nearly identical findings of embedded stones and papillary varices were discovered on the left. These were treated with laser unroofing and fulguration, respectively, with resolution of her symptoms once again.

## Discussion and conclusion

Varicose veins of the renal papillae have previously been described as the underlying etiology of chronic unilateral hematuria. Historically, diagnosis of such entities required radical or partial nephrectomy with subsequent pathologic examination. With the advent of endoscopic urology techniques, namely ureteroscopy, both the diagnosis and treatment of chronic unilateral hematuria was advanced such that the urologist might identify the source of bleeding in vivo. Thus, the categorization of diffuse and discrete lesions could be made more easily. Amongst the discrete lesions are MVR, which has been used as a catch-all term encompassing renal papillary varicosities. Not only was diagnosis advanced with modern endourologic techniques, but treatment was also revolutionized as surgical resection gave way to electrode fulguration and most recently laser ablation. Endoscopic techniques, predominantly flexible ureteroscopy with fulguration or laser ablation, have been shown to resolve 93% of cases of chronic unilateral hematuria with only a 10% rate of recurrence [2].

No prior case reports have demonstrated an association between nephrocalcinosis and renal papillary varicosities. Unique to this patient are the use of zonisamide and recent pregnancy prior to symptom onset. The underlying cause of renal stone formation related to zonisamide use is the inhibition of carbonic anhydrase, which leads to a renal tubular acidosis; however, rates of renal calculi formation with zonisamide are lower compared to those of other anti-epileptics, like topiramate and acetazolamide [3]. Pregnancy is known to cause renal physiologic changes that both promote renal calculi formation, such as hypercalciuria, but also counter-act it, like an increase in total urinary volume. But pregnancy also leads to systemic and renal vasodilatory changes. During pregnancy hormones such as RAAS, relaxin, and progesterone are all upregulated leading to vasodilatory effects and increased circulating blood volume [4]. This vasodilation could conceivably promote the development of renal papillary varices. The combination of these dilated blood vessels and inflammation from eroding renal calculi may explain the new onset and continuation of gross hematuria following her pregnancy.

Renal papillary varicose veins are an underlying cause of chronic unilateral hematuria. On one hand, work-up for discrete vascular lesions of the renal collecting system such as varices may be obscured by other benign co-existing conditions, such as nephrocalcinosis and nephrolithiasis in this case. On the other hand, a confluence of these conditions may in fact be the source of the patient’s symptoms. Regardless, flexible ureteroscopy with laser fulguration offers an ideal diagnostic and therapeutic modality for symptomatic renal papillary varicosities.

## Data Availability

Data sharing is not applicable to this article as no datasets were generated or analyzed during the current study.

## Abbreviations

CVA: Costovertebral angle
CT: Computed tomography
Fr: French
MVR: Minute venous rupture
RAAS: Renin-angiotensin-aldosterone system
RBC: Red blood cells
